# Exploring the quality of life of adolescents with Cerebral Palsy participating in conductive education around the Pannonian Basin

**DOI:** 10.1371/journal.pone.0277543

**Published:** 2022-12-01

**Authors:** Dóra Mladoneczki-Leszkó, Rebeka Surányi, Anna Kelemen

**Affiliations:** 1 Military Hospital- Medical Centre, Hungarian Defense Forces, Budapest, Hungary; 2 Semmelweis University—Doctoral School of Mental Health Sciences, Budapest, Hungary; 3 Eötvös Loránd University, Budapest, Hungary; 4 National Institute of Mental Health, Neurology and Neurosurgery, Budapest, Hungary; 5 András Pető Faculty, Semmelweis University Budapest, Hungary; Chiang Mai University, THAILAND

## Abstract

**Introduction:**

Quality of life (QoL) is a concept that includes physical, psychological, social, spiritual, and other domains of functioning. Good QoL is a fundamental goal of treatment for Cerebral Palsy (CP), therefore it is an outstanding goal of Conductive Education (CE) as well. CE is a Hungarian method that combines special education and rehabilitation for people living with CP.

**Objective:**

We aimed to compare the opinions and views about the life of teenagers from the perspective of adolescents and their caregivers from different socio-cultural backgrounds.

**Method:**

It was a descriptive, cross-sectional study. We used the Cerebral Palsy Quality of Life for the adolescent questionnaire (CP QoL -Teen) to measure QoL, which was translated into Hungarian and validated by Semmelweis University in 2017. Our study included 20 young adolescents (mean age 16) with CP and their caregivers living in Hungary (n_1_ = 40) and 20 Hungarian-speaking families (n_2_ = 40) from surrounding countries: Slovakia, Romania, and Ukraine (mean age 14.5). All the families are participating in CE.

**Results:**

There was no significant divergence in the whole QoL score between the groups. Nonetheless, we found an outstanding difference in the Hungarian groups’ ‘Feelings about functioning’ domain between teens and caregivers. A significant proportion of Hungarian teens–although living with greater pain–are less concerned about their illness (R = -0.754). 85% of responders study at segregated schools offering CE.

**Conclusion:**

The study shed new light on the importance of a personalized form of education and on the weight of the positive effects of segregated education. Personalized education can develop the patients’ QoL.

## Introduction

Cerebral palsy (CP) is the most common cause of neurodevelopmental disability. It affects the development of movement and posture, causing activity limitations attributed to non-progressive disturbances that occurred in the developing fetal or infant brain [1]. Besides the main motor symptoms, there are other disabilities as well: disturbances in communication, cognition, and psychological problems [2]. Furthermore, they may have problems with emotional relationships, and social factors may affect their behavior modification, too. There are three neuromotor types of CP: spastic, dyskinetic, and ataxic CP [3, 4]. There are also mixed types of CP. The main motor characteristics of spastic CP are spasticity and postural alignments, and there are some associated impairments, too (exp. perceptual problems, feeding problems, epilepsy, cognitive deficits, sensory loss, etc.) [5]. Involuntary movement, postural control problems, hypertonia, and palsy of gaze movements generally characterize dyskinetic CP. In this type of CP, the associated impairments are articulatory speech difficulties, specific high-frequency hearing loss, and emotional lability, but the patients’ level of intelligence is often good, and occasionally very high [6]. The main motor attributes of ataxic CP are disturbances of balance, hypotonia, and nystagmus. Intellectual impairment may exist in this kind of disability, especially regarding non-verbal cognitive skills [3–6]. The population of people with CP is heterogeneous [5]. Different individual impairments and dysfunctions can contribute to diminished participation in daily life [7]. A combination of the previously mentioned symptoms influences the quality of life of teenagers living with CP. Due to the many symptoms, it is difficult to research the QoL of CP, and it is a particularly neglected area in Hungary.

Conductive education is a pedagogical rehabilitation method. The goal is to develop an orthofunctional personality in different life domains, which means to be as close to normality as possible in the development of cerebral functions, acquiring the ability to live independently by developing proper motor coordination, and incorporating learned motor patterns automatically [8]. There are several forms of CE, depending on the infrastructural implementation. In Hungary, there is a special CE kindergarten and school, but in the surrounding countries, only interval CE is available. It means 2–4 weeks of CE per half-year besides regular school. One of the main goals of CE is to improve QoL in CP, as it is a lifelong disorder without a cure. The motor functions play a key role in self-care, and independence in self-care is another major goal of CE [9]. In CE, and in teenager groups, the Cerebral Palsy Quality of Life Questionnaire for Adolescents (CP QoL -Teen) should be an obligatory assessment tool to measure adolescents and their caregivers’ holistic well-being. We get feedback in the conductive groups about CP QoL -Teen, it gives a picture of how individuals feel about their own life. It is essentially subjective; it contains individuals’ judgment, thoughts, feelings, and values. CP QoL has received much attention over the last two decades in the wider world, and until now, Hungary has been an exception to this. Most studies have only focused on the parent-proxy version of the questionnaire, or used it as an equal questionnaire with the self-report one. Our knowledge of CP QoL -Teen in Hungarian-speaking families is largely based on limited data. The aim of our research was therefore to find differences between the parents-proxy and self-report versions of CP QoL -Teen, to focus on the opinions of families about QoL, and to evaluate its socio-cultural and organization-specific aspects. According to our hypothesis, there is a linear relationship between the answers given by parents and children to the same question; one of the aims of the research is to confirm or disprove this linear relationship between the answers of parents and their children of the same residence, and parents or children of different citizenships.

## Materials and methods

### Design

This was a descriptive, cross-sectional study.

### Participants

Families were eligible to participate in this study if they had a child with a confirmed diagnosis of CP aged 13 to 18, participating in CE (followed up for at least 5 years). Another criterion was that the teenager can fill the questionnaire independently or with little help from the conductor. Eligible families were identified through the Semmelweis University Pető András Faculty (n = 40 families). Forty adolescents with CP and 40 primary caregivers completed the questionnaire. The primary caregiver was identified as the person who knew the most about the child; in our study they were all parents of the teenagers.

Among the calculated Pearson correlation coefficients, relationships with an effect size of at least 0.6 were taken into account. The minimum number of subjects required for this research is 19, in addition to the generally accepted 0.05 (type I error) and 0.2 (type II error), which was ensured in this research.

### Data collections

Primary caregivers completed the CP QoL -Teen; parent-proxy version and questionnaire about the socio-cultural milieu. Adolescents completed the CP QoL -Teen (adolescent self-report version) for children aged 13 to 18 years. Medical and functioning data (type of CP, gross motor function, manual ability, communication function) were collected from conductors’ documentation. The participants were broken down into two groups based on their country of residence: the Hungarian group living in Hungary and the Pannonian Basin group living in surrounding countries, but speaking Hungarian within their families. Data were collected first in Hungary, and afterwards in the surrounding countries.

### Statistical analyses

Statistical significance was analyzed using the IBM SPSS Statistics 22 software. According to the initial hypothesis, there is a linear relationship between the answers given by parents and children to the same question, one of the aims of the research is to confirm or disprove this linear relationship between the answers of parents of the same nationality and their children, as well as parents or children of different nationalities. The most suitable method for this is Pearson’s correlation. Pearson’s correlation coefficient was used to reveal the correlations between the answers to each item of the scale within the 5/7 domain scale. This is the most appropriate measure to express the strength of the linear relationship between two variables. It is required to test all the individual variable pairs’ linear distribution to calculate the Pearson correlation. In our study, we used a scatter plot for validating the prerequisite linear correlation between variable pairs labeled strongly correlating. The line of the linear regression model is marked on the figures along with the data set and its correlation to the linear regression model’s line. According to the regola practica (rule of thumb) if the R2 linearity value is 0,3 or higher the data could be considered linear (S1 File).

The scores of the answers given for each domain were averaged according to the CP QoL manual. We examined the possibility of a difference between the averages of the answers of Hungarian parents and their teens, Pannonian Basin parents and their teens, Hungarian and Pannonian Basin parents, and Hungarian and Pannonian Basin adolescents, by using the two-sample t-test. Within each domain, Principal Component Analysis was performed, and then a two-sample t-test was applied to the established principal components to find out if there was a difference along the established dimensions between the groups.

### Quality of life questionnaire

CP QoL -Teen was developed by an international team of clinicians and researchers at The University of Melbourne and the Royal Children’s Hospital. CP QoL -Teen has been developed for adolescents aged 13–18. It has been translated into the following languages: Arabic, Bengali, Brazilian-Portuguese, Burmese, Chinese-Cantonese, Dutch, Greek, Hungarian, Italian, Japanese, Malayalam, Russian, Spanish, and Catalan. In our study, we used the Hungarian type of questionnaire, which was translated into Hungarian and validated by Semmelweis University in 2017 [10]. There are two different types of questionnaires: the adolescent self-report version, comprising of 72 items, and the parent-proxy version, containing 88 items. The CP QoL -Teen questionnaire has acceptable reliability and emerging evidence of validity [11, 12]. CP QoL -Teen parent-proxy version measures seven areas of an adolescent’s life: general well-being and participation, communication and physical health, school well-being, social well-being, feelings about functioning, access to services, and family health [13]. CP QoL -Teen parent-proxy version measures seven areas of an adolescent’s life: general well-being and participation, communication and physical health, school well-being, social well-being, feelings about functioning, access to services, and family health [14, 15]. The self-report version assesses all of the above domains except access to services and family health. Almost all of the items have the following item stem: ‘How do you think your teenager feels about…?’ with a nine-point rating scale, where 1 = very unhappy, 3 = unhappy, 5 = neither happy nor unhappy, 7 = happy, and 9 = very happy [11].

### Functional measures of adolescents living with CP

Functioning data were collected from conductors. The classifications delineate and clarify the levels of function in cerebral palsy. Each of the classification systems uses five levels, where level V represents individuals with severe problems. The Gross Motor Function Classification System (GMFCS) describes the level of functional ability, in a developmental picture, using age‐dependent criteria. Each level is based on a concise description, indicating what an adolescent can do in self‐initiated motor functions such as sitting, changing postures, crawling, standing, and walking [16]. Manual Ability Classification System (MACS) explains a teenager’s ability in self‐initiated hand functions at home, school, or in community. The MACS and GMFCS classifications do not describe the levels of skills [17]. Communication Function Classification System (CFCS) classifies levels of everyday communication efficiency. Every procedure of communication is considered such as speech, gestures, behaviors, eye gaze, facial expressions, and augmentative and alternative communication (manual sign pictures, talking devices, and communication boards [18, 19].

### Procedure

The primary caregiver was invited to take part in this investigation by their teenager’s conductor (in person or in writing). The lack of Hungarian language proficiency did not constitute a reason for exclusion, as interpreters were available although not required. We asked for written informed consent from the primary caregiver and patients. If they consented, the questionnaires were sent by e-mail or were mailed. The teenagers filled out the questionnaire under the guidance of a conductor who did not know them, but had a vast experience in helping children fill in the form without influencing their decisions. Teenagers who could not complete the questionnaire due to their intellectual impairment or inability to communicate were excluded from the study, and so were their parents. Ethical approval was obtained from Semmelweis University (SE 10793163, IKT: 54891/ PAKDH/ 2020).

## Results

### Demographic characteristics

As demonstrated in Table 1, adolescents were aged between 13 and 18 years in both groups. Most of the juveniles in both groups have spastic CP (75%). The adolescents’ motor function levels were determined using the Gross Motor Functional Classification System (GMFCS Level I = 12, II = 9, III = 7, IV = 8, V = 4) and Manual Ability Classification System (MACS Level I = 11, II = 15, III = 8, IV = 5, V = 1). The everyday communication effectiveness level was determined by Communication Function Classification System (CFCS Level I = 20, II = 9, III = 8, IV = 3). The levels indicated that the adolescent in the study covers the spectrum of motor subtypes and motor disabilities. In both groups, the teens’ average CFCS Level is I.

**Table 1:** Demographic characteristics.

Adolescent					
		*Hungarian (n = 20)*		*Pannonian basin (n = 20)*	
		Frequency	Percent	Frequency	Percent
**Age (range, mean)**		13–18 (range)	16 (mean)	13–18 (range)	14.5 (mean)
**Gender**	Male	11	55%	11	55%
	Female	9	45%	9	45%
**GMFCS levels**	I.	4	20%	8	40%
	II.	3	15%	6	30%
	III.	3	15%	4	20%
	IV.	6	30%	2	10%
	V.	4	20%	0	
**MACS levels**	I.	3	15%	8	40%
	II.	9	45%	6	30%
	III.	4	20%	4	20%
	IV.	3	15%	2	10%
	V.	1	5%	0	
**CFCS levels**	I.	10	50%	10	50%
	II.	3	15%	6	30%
	III.	5	25%	3	15%
	IV.	2	10%	1	5%
**Neuromotor Classification**	Spastic	15	75%	15	75%
	Dyskinetic	4	20%	2	10%
	Ataxic	1	5%	3	15%
**Education**	Special school	17	85%	10	50%
	Elementary school	2	10%	8	40%
	Secondary school	1	5%	2	10%
**Primary caregiver**					
		***Hungarian (n = 20)***		***Pannonian basin (n = 20)***	
		**Frequency**	**Percent**	**Frequency**	**Percent**
**Residence**	Urban	12	60%	14	70%
	Rural	8	40%	6	30%
**Mothers’ education**	Less than a high school diploma	8	40%	5	25%
	High school degree or equivalent	7	35%	5	25%
	Bachelor’s degree (e.g. BA, BS)	3	15%	6	30%
	Master’s degree (e.g. MA, MS, MEd)	2	10%	4	20%
**Fathers’ education**	Less than a high school diploma	8	40%	6	30%
	High school degree or equivalent	5	25%	4	20%
	Bachelor’s degree (e.g. BA, BS)	2	10%	8	40%
	Master’s degree (e.g. MA, MS, MEd)	3	15%	2	10%
	Doctorate (e.g. PhD, EeD)	2	10%	0	

Values are n (%) or mean (SD). GMFCS- Gross Motor Functional Classification System, MACS- Manual Ability Classification System, CFCS- Communication Function Classification System.

To evaluate the cognitive abilities of CP Teen, we used the school studies as a basis, and asked the conductors about their professional opinion about adolescents. Although 85% of the Hungarian group studies in a segregated institution, they have a regular education plan in the school supplemented with conductive lessons.

Most parents from Hungary had less than a high school diploma: 40% of mothers and 40% of fathers. The majority of Pannonian Basin parents have completed bachelor’s degrees: 30% of mothers and 40% of fathers. Our group can be considered homogeneous with minor variations; In such a heterogeneous disease, this is still acceptable according to our statistician.

### Differences between adolescents’ and parents’ answers in Hungary

When comparing the responses of Hungarian parents and their teenage children, we found that the highest ratio of significant correlations was detectable regarding the School well-being domain (40%, Table 2). The most prominent relation was observed in the Social well-being domain, where the majority of respondents rated the same: spending their leisure time with their family (R = 0.868, (S1A Table in S1 Data)). On average, we found a strong positive correlation between the answers that involve acceptance: how well adults receive teenagers living with CP (R = 0.864 (S1A Table in S1 Data)), resp. acceptance of other students in the school (R = 0.849 (S1A Table in S1 Data)). The General well-being and School well-being domains showed the lowest ratio of significant correlations between the opinions of parents and their children (Table 2).

**Table 2:** Differences between adolescents’ and parents’ domains in Hungary.

CP QoL domain	Number of questions	Number of significant correlations	Rate of significant correlations
*General well-being & participation*	21	115	26%
*Communication & physical health*	17	67	23%
*Social well-being*	7	6	12%
*School well-being*	9	32	40%
*Feelings about functioning*	14	68	35%
*Access to services*	teen:4, parent:16	1	2%

The highest discrepancy between parents and teens was found in the items ‘concerned about having cerebral palsy’ and ‘how much pain she/ he has’. A significant proportion of Hungarian teens rate CP as not worrying, but the parents gave considerably higher scores for this item. Adolescents taking part in CE, although reported living with greater pain, are less concerned about their illness, which means that they do not identify the pain with CP (R = -0.754 (S1A Table in S1 Data)). The pain item in the questionnaire is not defined, it could be emotional, psychical, or physical pain, or a combination of these.

### Differences between adolescents’ and parents’ answers in the Pannonian basin

In the Pannonian Basin group (Table 3), based on the feedback from the parent and their teen children, the most significant correlations were observed in the School well-being (72%) and the Communication and physical health domains (51%). The three strongest relationships were found in the Communication and physical health domain.

**Table 3:** Differences between adolescents’ and parents’ domains in the Pannonian basin.

CP QoL domain	Number of questions	Number of significant correlations	Rate of significant correlations
*General well-being & participation*	21	198	45%
*Communication & physical health*	17	148	51%
*Social well-being*	7	13	27%
*School well-being*	9	58	72%
*Feelings about functioning*	14	39	20%
*Access to services*	teen:4, parent:16	3	5%

The highest correlation accuracy between patients and caregivers was R = 0.958 (S1B Table in S1 Data) in the item about how a CP teen communicates with other, less close acquaintances. The way the adolescent communicates with close friends is related to the way he/she communicates with less close friends (R = 0.945(S1B Table in S1 Data)). There was a discrepancy between the Pannonian Basin parents who found it difficult to access respite care in their dwelling place and the teens who were satisfied with the relief equipment available to them and experienced (R = -0.882 (S1B Table in S1 Data)).

### Differences between parents from Hungary and the Pannonian Basin

The opinions of Hungarian and Pannonian basin parents were very different (Table 4); the highest significant correlations ratio was in the General well-being and participation domain, which was only 8%. Interestingly, there was no consequential correspondence at all in the Social well-being and School well-being domains (0%). The three strongest correlations were found in the Communication and physical health domain, which means that they have the same opinions on the adaptation of their adolescents. The highest inverse correlation was measured in the General well-being and participation domain, where the positive attitude of the teen in the Pannonian Basin is extremely good according to their parents, unlike the attitude of teens in Hungary, which was confirmed by their parents saying that the satisfaction of the teenagers with their life is generally negative (R = -0.626 (S1C Table in S1 Data)).

**Table 4:** Differences between parents’ domains from Hungary and Pannonian basin.

CP QoL domain	Number of questions	Number of significant correlations	Rate of significant correlations
*General well-being & participation*	21	36	8%
*Communication & physical health*	17	11	4%
*Social well-being*	7	0	0%
*School well-being*	9	0	0%
*Feelings about functioning*	14	5	3%
*Access to services*	16	8	3%
*Family health*	5	1	4%

### Differences between adolescents from Hungary and Pannonian-based areas

The responses of Hungarian and Pannonian basin teenagers also rarely show a significant correlation (Table 5). The highest proportion of significant correlations was noticeable in the Social well-being domain (10%) with no similarity, and totally different ratings in the School well-being and Access to services domains. For both domains, the Hungarian teen population gave a more positive assessment. The strongest positive correlation was between the items ‘going on trips with the family’ and ‘the way the adolescents get along with their parents’ (R = 0,840 (S1D Table in S1 Data)).

**Table 5:** Differences between adolescents’ domains from Hungary and Pannonian-based areas.

CP QoL domain	Number of questions	Number of significant correlations	Rate of significant correlations
*General well-being & participation*	21	8	2%
*Communication & physical health*	17	4	1%
*Social well-being*	7	5	10%
*School well-being*	9	0	0%
*Feelings about functioning*	14	15	8%
*Access to services*	4	0	0%

## Discussion

The present study is the first to compare QoL between the Hungarian and Pannonian Basin CP populations participating in CE. Some aspects were already explored in the QoL of children with CP living in Hungary, but the population of teenagers with CP was not examined yet. As we know, different levels of movement, orthopedic, and cognitive impairments are common in individuals with CP [5, 7]. The teenagers who live with CP have different levels of mobility and self-sufficiency, which makes their social integration difficult [20]. Milićević reports that fine motor skills, home adaptation, and the magnitude of barriers predicted physical well-being [21]. Traditional medical treatment focuses primarily on the physical symptoms of patients, while several studies demonstrate that the level of functioning (measured by GMFCS, MACS, and CFCS) is an independent predictor of impairment in QoL in children with CP [22–24]. Our group was relatively homogenous regarding the functional status of the children and regarding non-medical parameters, such as the education level and the income of the family. We examined whether different country of residence can influence QoL under similar educational and economic circumstances. Although there are researches that provide information from the caregivers’ perspective, home adaptation, supportive laws and policies, and family-centered rehabilitation care may promote children’s QoL if they meet the families’ needs [21]. Stocker et al. confirmed that parents and children are more likely to have lower perceptions of both individual and family QoL, which were mainly associated with psychological factors, such as low self-esteem, chronic stress, anxiety, and depression. These could have negative repercussions on parental health as well [25].

Based on this knowledge, we have hypothesized that different socio-cultural backgrounds may influence CP QoL, and judgments of QoL may differ between parents and their teenage children. It was noteworthy that in both the Hungarian and the Pannonian Basin groups, there was an agreement between adolescents’ and their primary caregivers’ opinions. It can be seen that the scores given by the parents in both groups were similar to the responses of the adolescents regardless of their country of residence. Analyzing the different domains of the questionnaire, some questions showed striking differences between the child and the parent. The most notable difference was found in Hungarian families, where the highest discrepancy between the parents and the teens was found in the items ‘concerned about having cerebral palsy’ and ‘how much pain she/ he has’(R = -0.754). A significant proportion of Hungarian teens rate CP as not worrying, but the parents gave a considerably higher score for the above item. This can probably be explained by the fact that 85% of Hungarian adolescents were from a segregated but homogenous conductive institution, where acceptance, specific education, and personalized treatments help the child to accept the condition, and the group effect works as a driving force. In segregated homogenous surroundings, the teens’ experience of CP does not look different, they do not consider their functional problem as a disease, and they all see themselves equal. In CE schools, such as the Pető Institute in Budapest, although each member of the group carries out the tasks at the level that suits them, the common problem of organizing tasks is always solved individually, even though group suggestion and social facilitation prevail [8, 9, 26]. A sense of belonging to the group and protection makes it easier for adolescents living with CP to express themselves, playing an important role in the development of self-esteem. The group can influence the development of the people who belong to it, and improve their self-discipline and self-control [27, 28]. Based on psycho-sociological research, we can say, that the happiness of an individual with a disability is subjectively higher in a socially supportive environment [29]. Csuka et al. found that the social anxiety scores were higher in integrated schools than in segregated ones. They believe that the moderately affected CP population is particularly vulnerable in this respect [30].

Both groups of surveyed parents evaluated all seven domains equally. The Hungarian and Pannonian Basin parent groups had little similarity, however, in both groups, parents gave the most negative scores to the Access to services domain. This critique was particularly prominent in the Pannonian Basin group. In this section, we were surprised to see that the Pannonian basin parents had largely different opinions from their children in terms of aids, various benefits, therapies, special equipment, and relief care (R = -0.882). Both the Hungarian and Pannonian Basin parent groups were the least satisfied with the relief care and its use. Surprisingly, despite all this, there was no dissatisfaction in either group in the Family health domain. Unlike other research carried out in this area, we did not find a significant difference between the socio-economic status of the family and the quality of life. According to some publications, long-term outcomes in different aspects of functioning were influenced by the family’s financial situation or the mother’s occupation [31]. Our study is consistent with previous results in adults, which suggest that quality of life is mainly influenced by psychological factors: interpersonal relationships, personal development, and social acceptance, rather than physical well-being or material well-being [32, 33]. The results of our study also show that the medical aspects of the disease and therapy do not influence QoL that much, and we conclude that other social factors have considerably higher importance. Since this is not a sociological study, we cannot determine what these factors are, as we did not examine them in detail, but the only comprehensible difference was in the organization of the way CE was delivered. In a segregated CE school form in Hungary, and an interval form in additional to regular schooling in surrounding countries. This difference might have a significant and relevant role. To get a comprehensive picture of adolescents’ QoL, it would be necessary to conduct studies from both the parents’ and the children’s, perspectives [31, 32]. In our research, the strength of correlations between adolescents’ and their parents’ opinions points to the fact that one questionnaire might be enough in a clinical setting. In our principal component analysis, we can see that the "vision for the future" and the positive attitude in the "General wellbeing & participation" domain show a significant difference between the Hungarian and Pannonian Basin groups (p = .004). The "vision for the future" of the Pannonian Basin teens shows a more negative picture than that of the Hungarian population, which may be because these children may face psychological burdens daily in an integrated environment contrary to segregated CE school teens. The reason is not to be searched in the acceptance and attitude of the teachers and schoolmates, since we got positive results in both dwelling places in this item. The reason may lie in the process–and we suppose that in the psychological aspects–of integration. The way adolescents feel about their physical differences in this very vulnerable age group and whether they can or cannot participate in sports and leisure activities can be decisive, considering that children with CP need greater social acceptance and support than their healthy peers.

### Strengths and limitations

The results of this study must be interpreted within the context of the sample. We are aware that our research may have limitations. The first one is the low number of participants. Unfortunately, it was not possible to collect more data, because several parents did not provide consent. Only 20 out of 70 families in the Pető Institute agreed to participate in the research. The second is we did not measure the IQ of the teens in this study, but all of them attended "traditional" education, so we suppose their IQ is within the normal range.

## Conclusions

The results indicate that adolescents with CP perceive their QoL almost identically to their parents. Together, these results suggest that interpersonal relationships, school well-being, social well-being, and communication were the most important factors for QoL for teens, whereas access to services and feelings about functioning were the least important ones. Self-acceptance showed higher value in the segregated environment, which suggests that more attention should be paid to the known factors that can lead to an increase in anxiety in an integrated environment (responsibility, success orientation, failure avoidance, development of cooperation skills). From the parents’ point of view, they experience the access to services as negative, regardless of their country of residence, which can be characteristic for the whole region. Parents are under severe mental, physical, and financial strain regardless of the country they live in. Based on our results, we believe that, as much as possible, efforts should be made to stimulate self-activity in the CP population, with particular focus on maintaining the initiative in some cases. Furthermore, it would be beneficial to provide an opportunity to build a community with a similar CP teen. Our work has some limitations. Still, we believe our work could be a springboard for finding factors affecting the QoL of the CP adolescent population. The present findings might highlight the positive views of a segregated institution, such as its protective role. It was planned to collect more data from different socio-cultural environments, but it has not been implemented yet due to the COVID-19 pandemic. To extend our research, we are planning to validate the results of this study with a larger sample size, including CP teens from other countries as well.

## Supporting information

S1 Data Correlations between different groups. **A:** Correlations between adolescents’ and parents’ answers in Hungary divided into domains. **B:** Correlations between adolescents’ and parents’ answers in the Pannonian Basin divided into domains. **C:** Correlations between parents from Hungary and Pannonian Basin divided into domains. **D:** Correlations between adolescents from Hungary and Pannonian Basin divided into domains.
(XLSX)

S1 File Pearson-correlation scatter plots. (PDF)
