# Supplementary material for: Exploring the quality of life of adolescents with Cerebral Palsy participating in conductive education around the Pannonian Basin
Source: PLoS One. 2022 Dec 1;17(12):e0277543. doi: 10.1371/journal.pone.0277543 (PMC9714917; doi:10.1371/journal.pone.0277543)
Supplement: S1 File — (PDF) [file pone.0277543.s002.pdf]

# *Pearson-correlation scatter plots*

Each question begins with 'How do you FEEL about.....?'or 'How do you think your teenager FEELS about.....?.'

### 1. *Differences between adolescents' and parents' answers in the Pannonian basin*

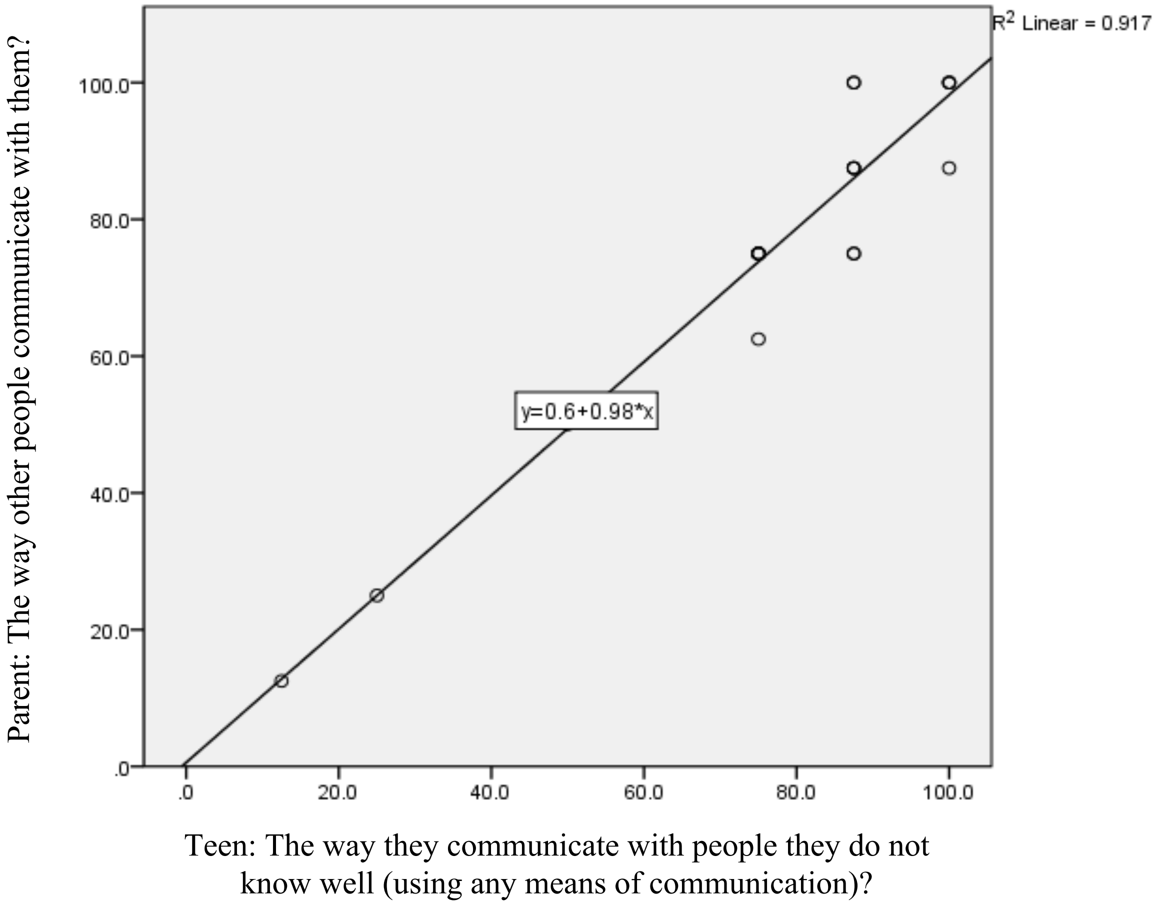

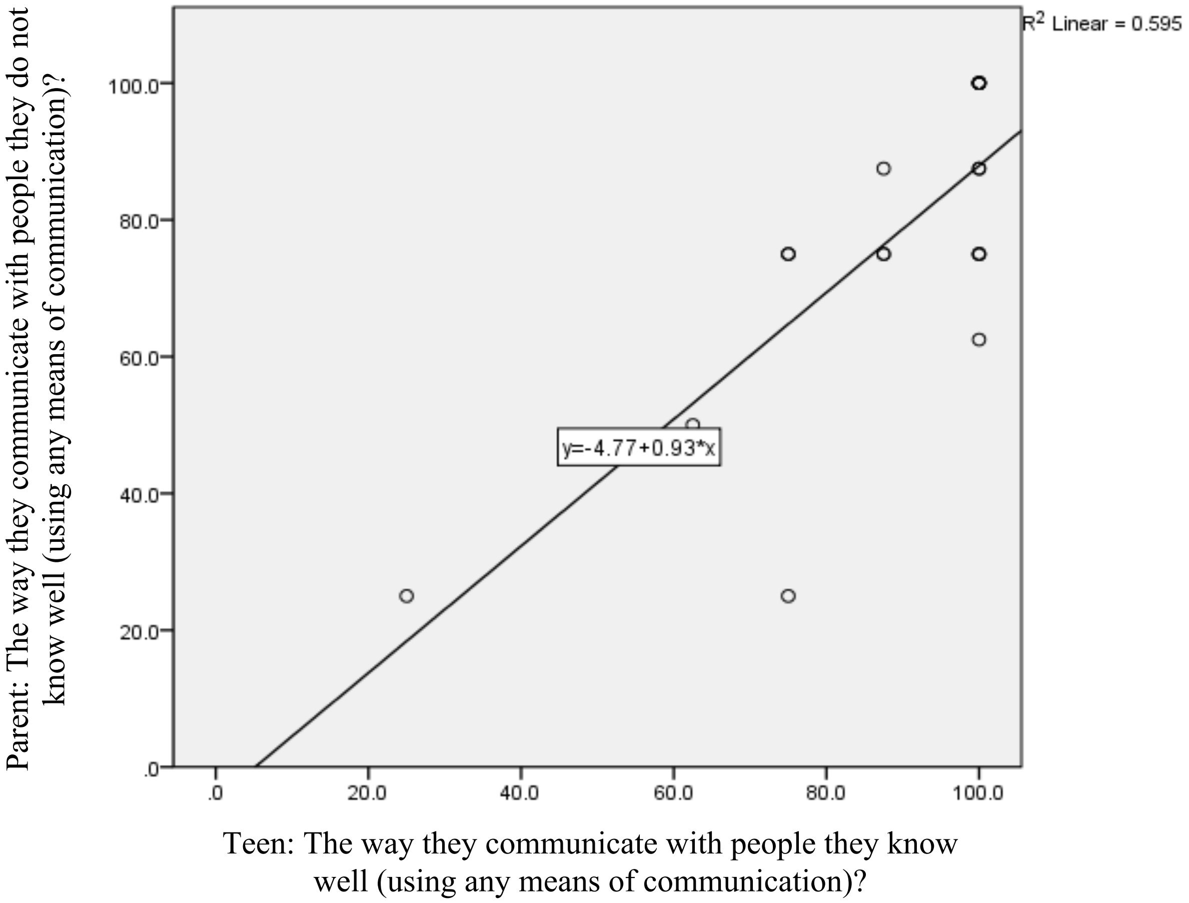

R2 Linear = 0.595
y=-4.77+0.93*x
100.0
80.0
60.0
40.0
20.0
.0
.0
20.0
40.0
60.0
80.0
100.0
Parent: The way they communicate with people they do not know well (using any means of communication)?
Teen: The way they communicate with people they know well (using any means of communication)?


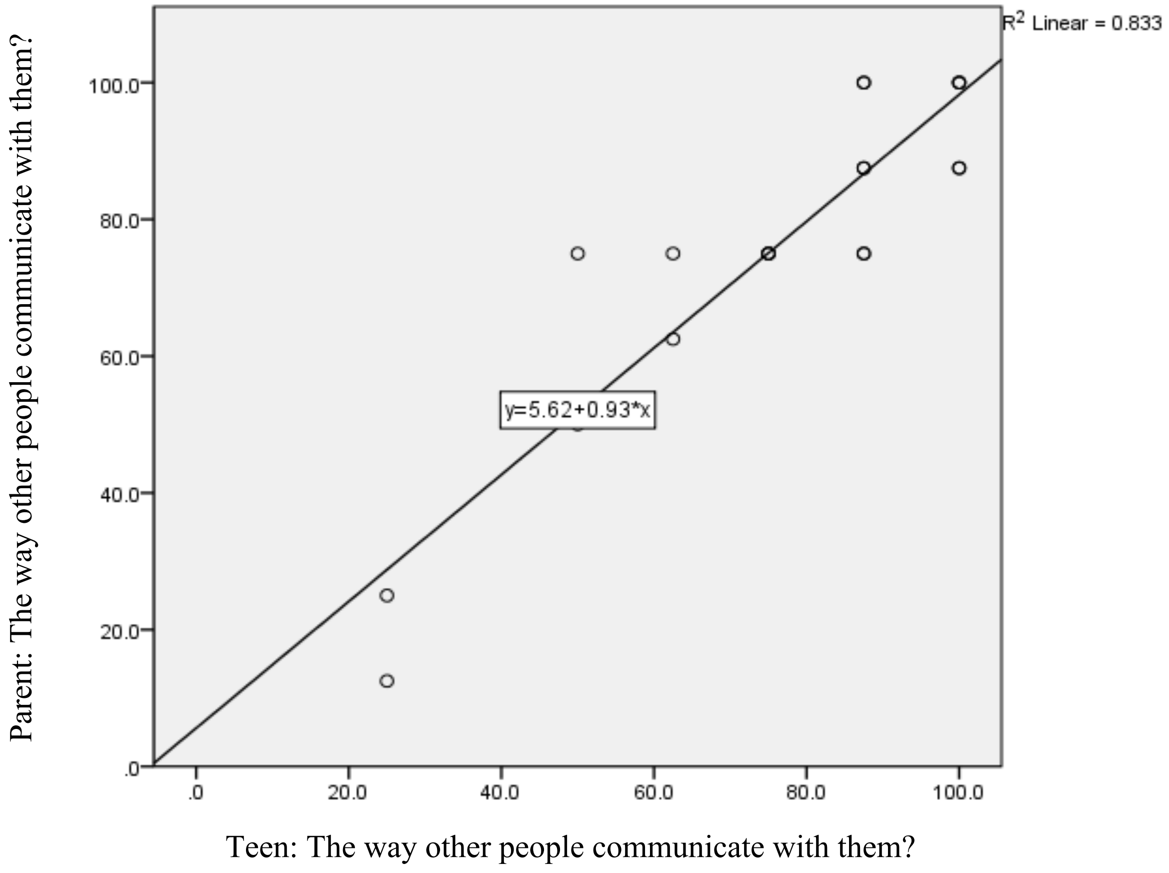

R2 Linear = 0.833
y=5.62+0.93*x
100.0
80.0
60.0
40.0
20.0
.0
.0
20.0
40.0
60.0
80.0
100.0
Parent: The way other people communicate with them?
Teen: The way other people communicate with them?

## *2. Differences between adolescents' and parents' answers in Hungary*

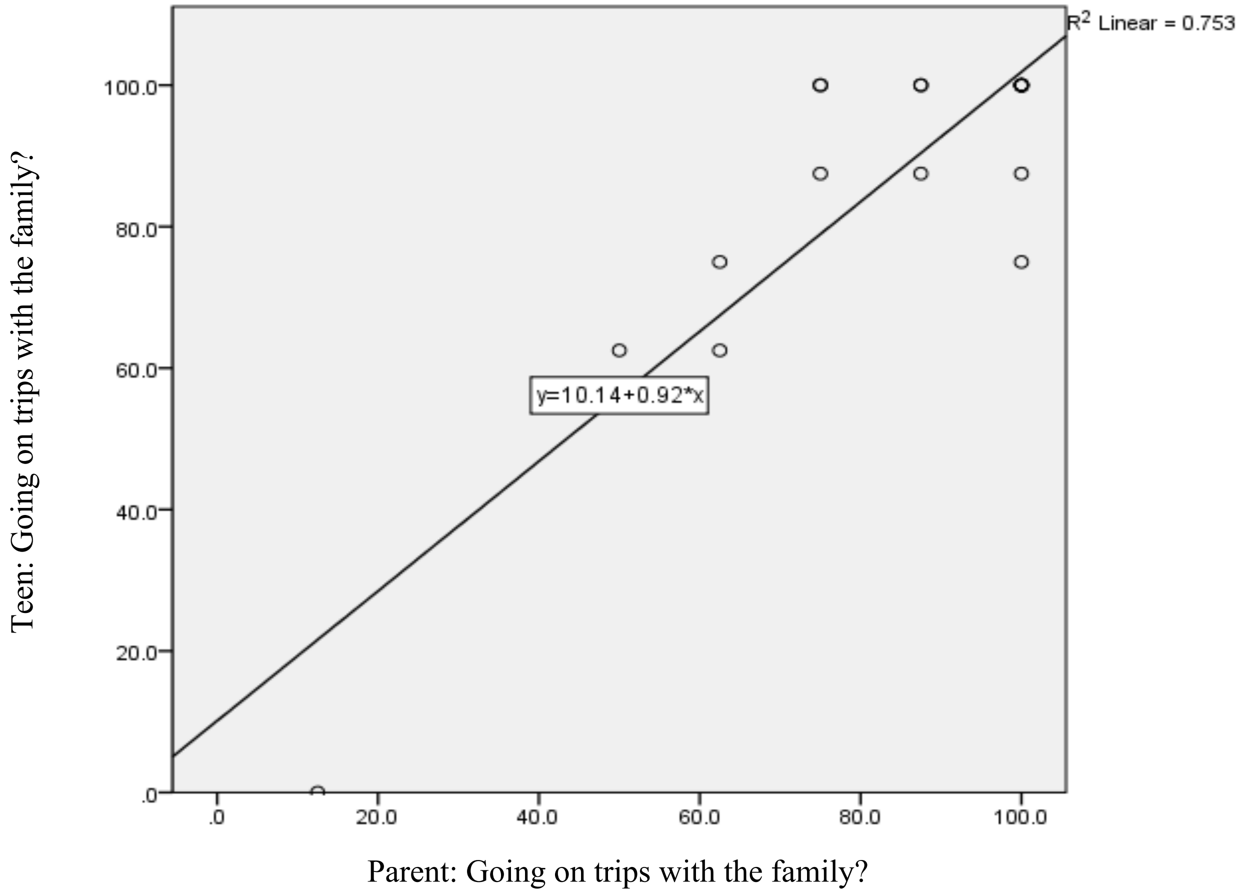

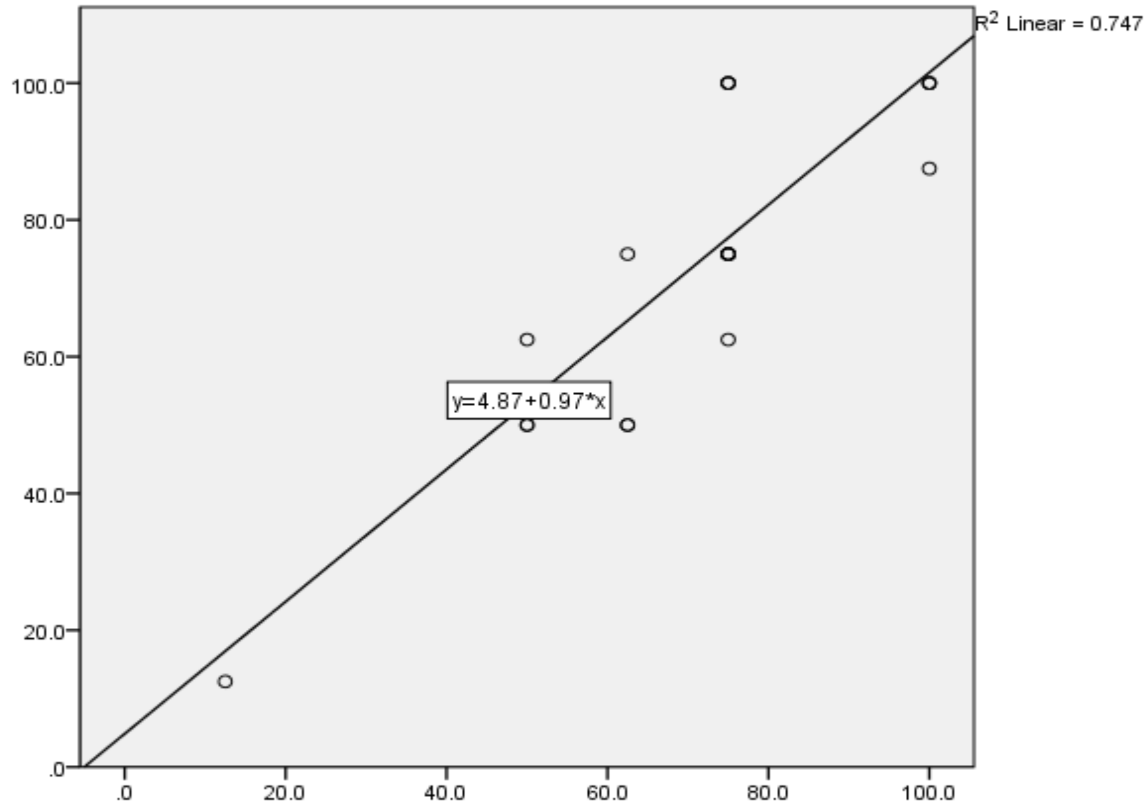


Teen: How you are accepted by adults?

Parent: How they are accepted by people in general?

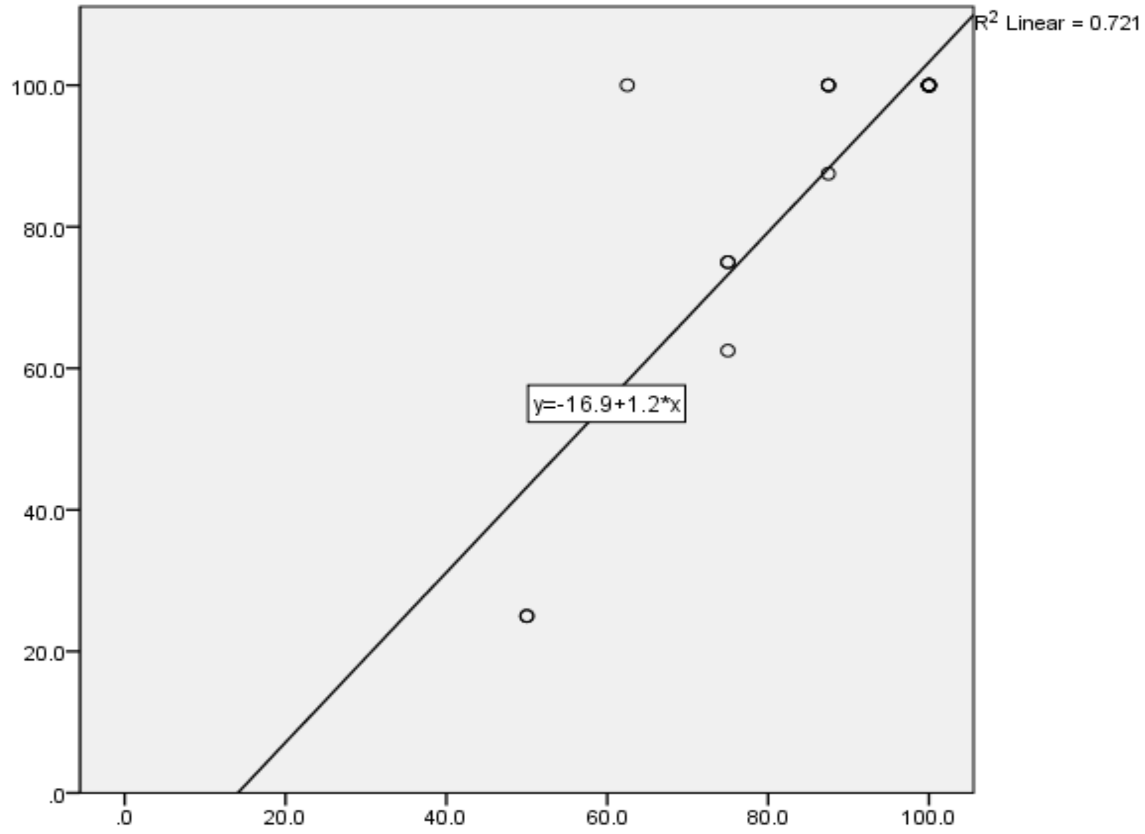


Teen: The way you are included by other students at your school?

Parent: How they are accepted by other students at school?

### *3. Differences between adolescents' from Hungary and Pannonian-based areas*

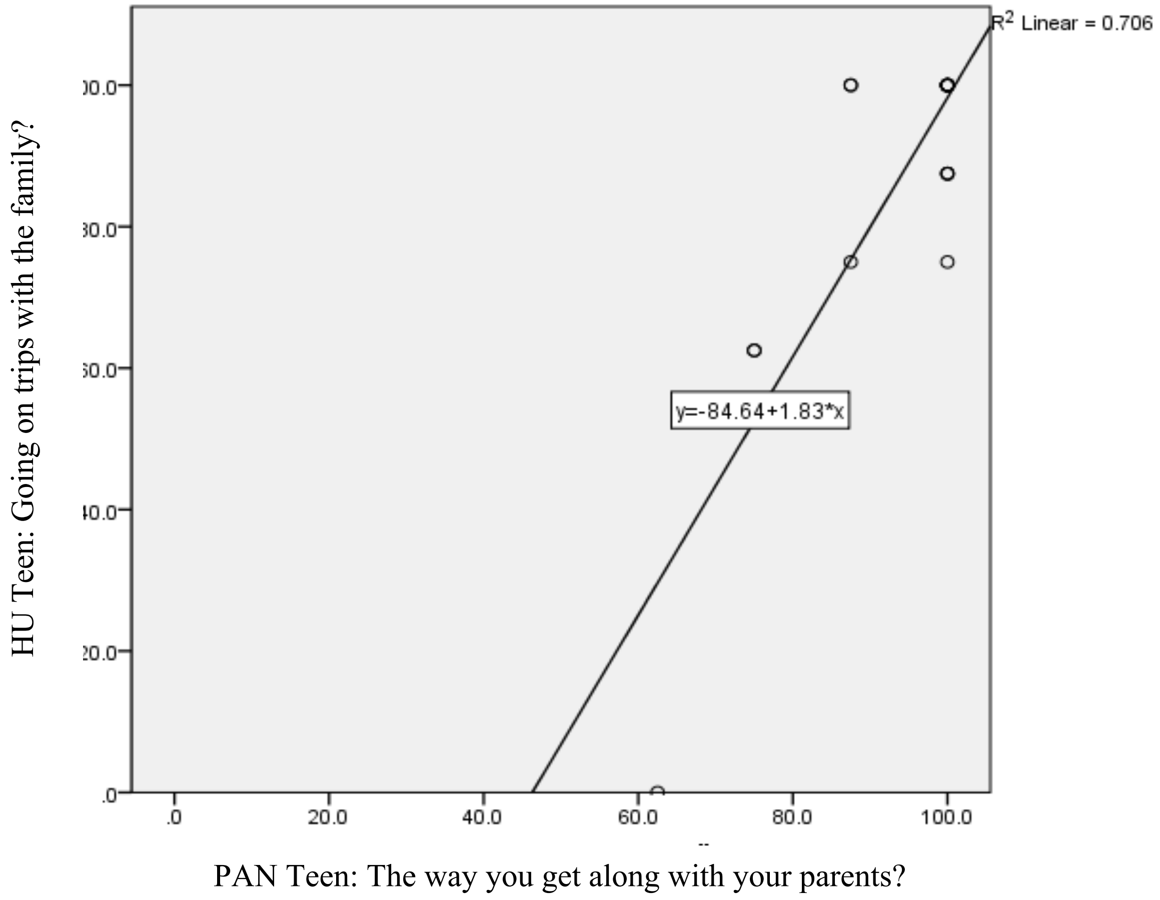

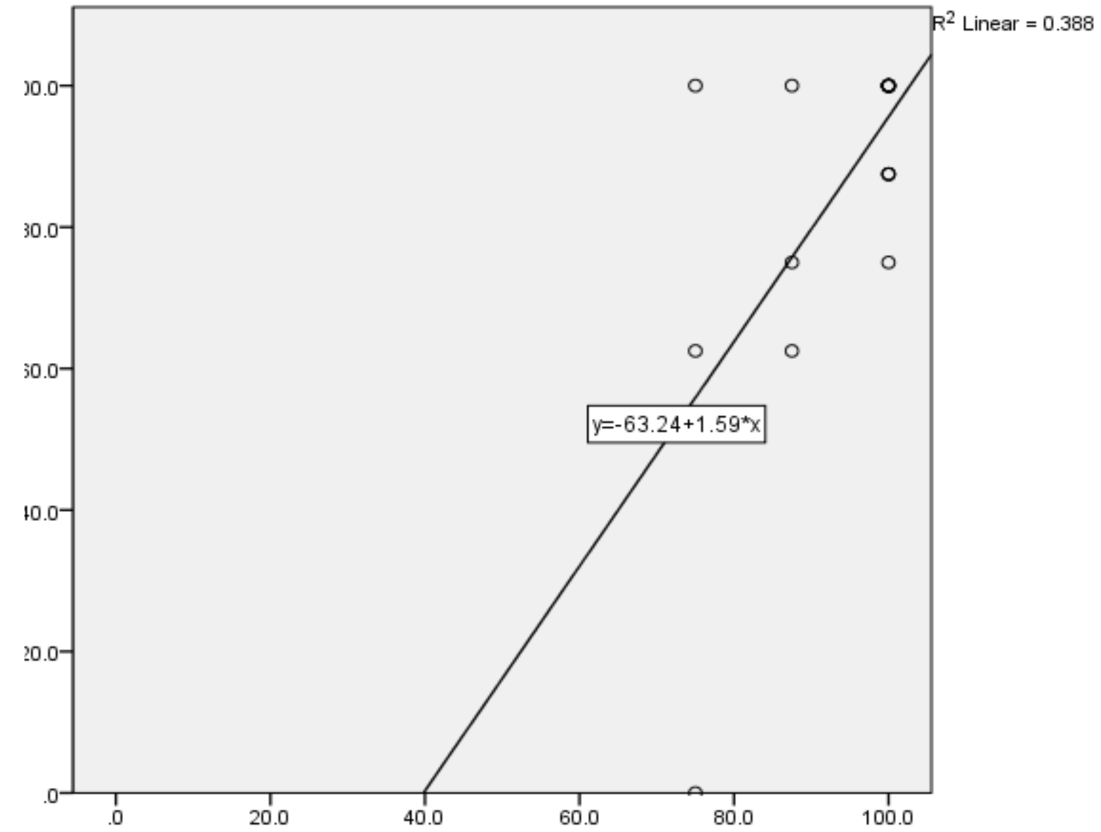


HU Teen: Going on trips with the family?

PAN Teen: The support they get from your family?

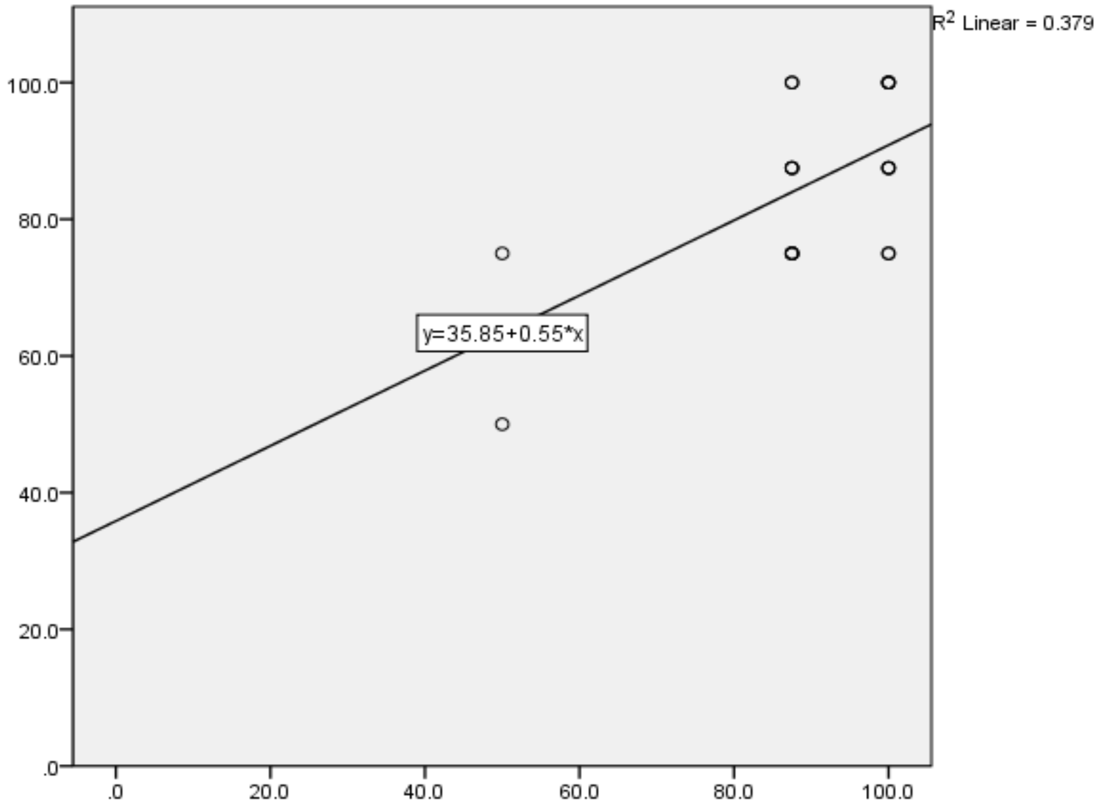


HU Teen: The way you get along with people generally?

PAN Teen: Goin gon trips with the family?

### 4. *Differences between parents from Hungary and Pannonian basin*

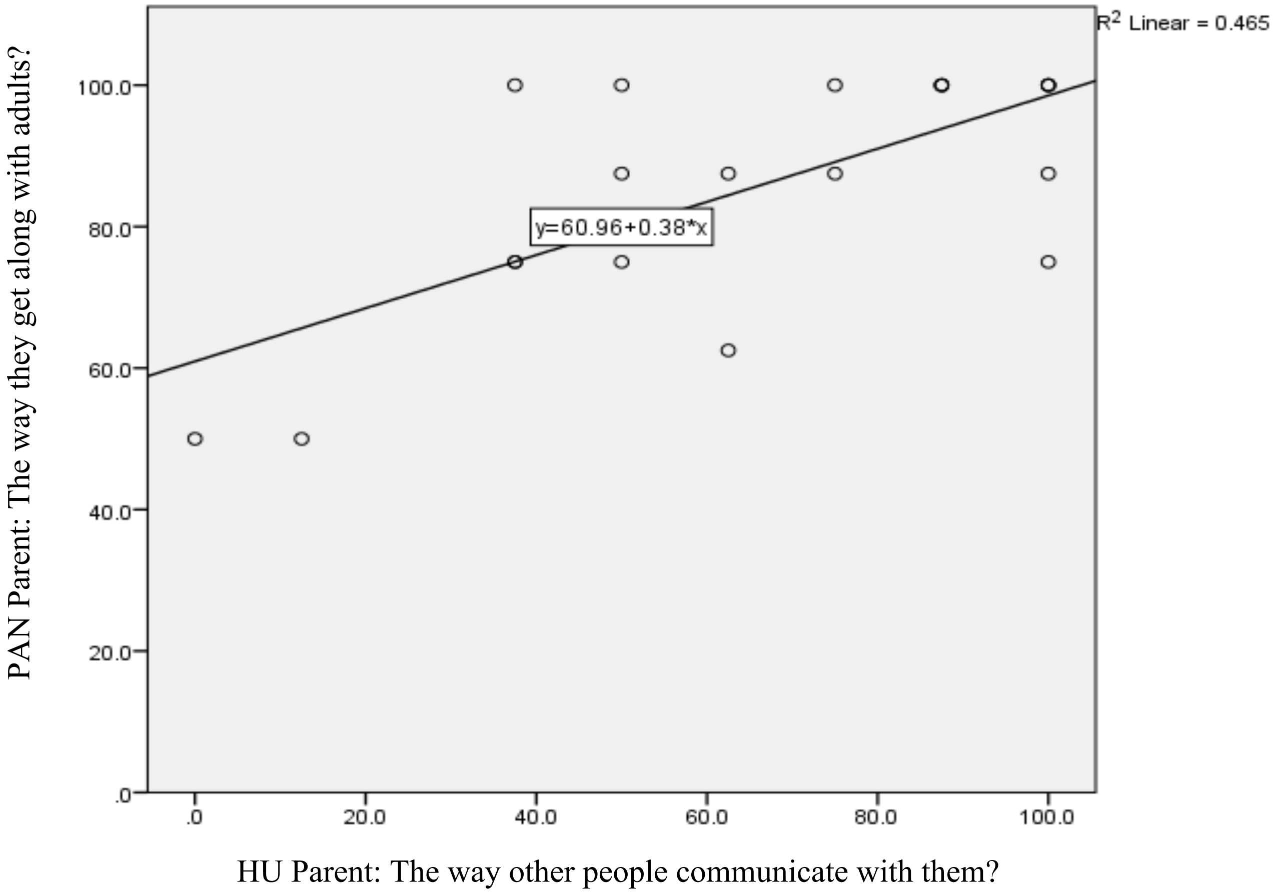

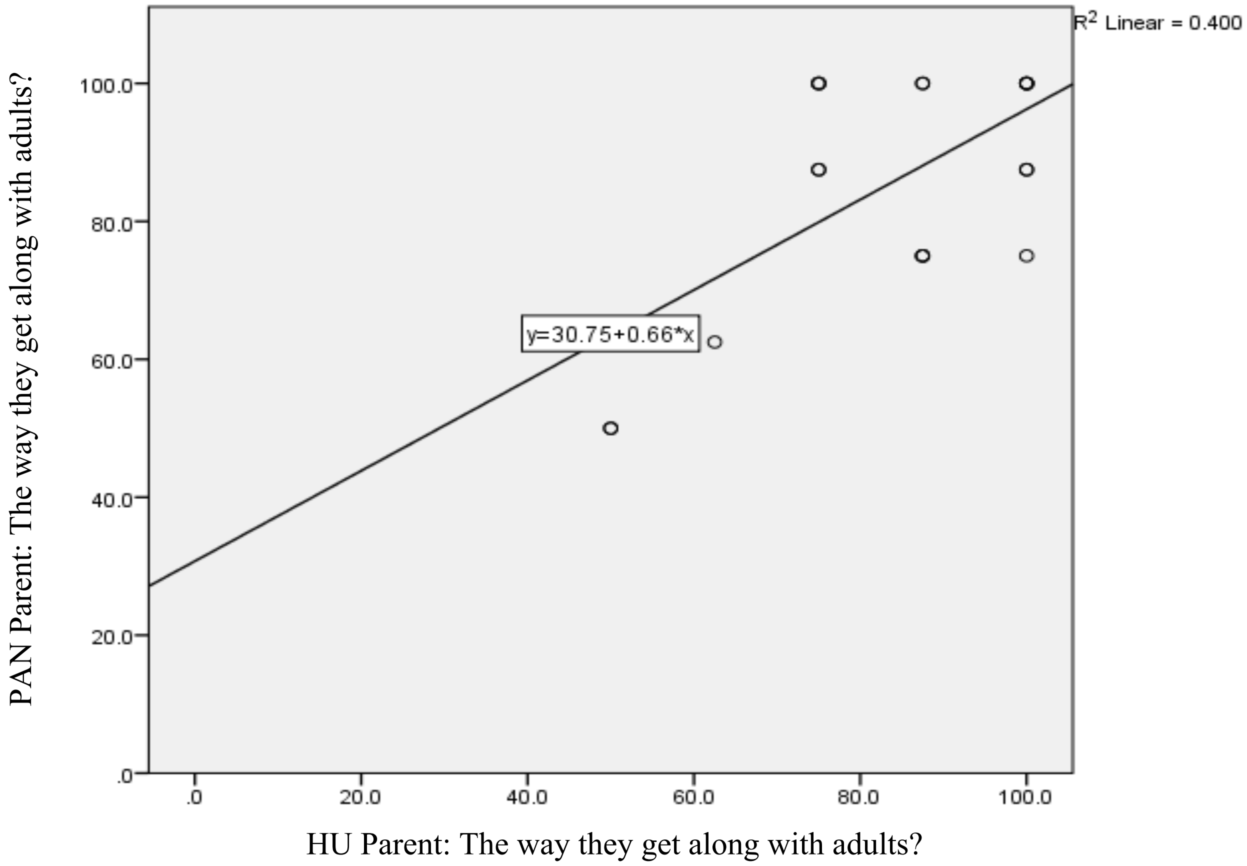
R2 Linear = 0.400
y=30.75+0.66*x
100.0
80.0
60.0
40.0
20.0
.0
.0
20.0
40.0
60.0
80.0
100.0
PAN Parent: The way they get along with adults?
HU Parent: The way they get along with adults?

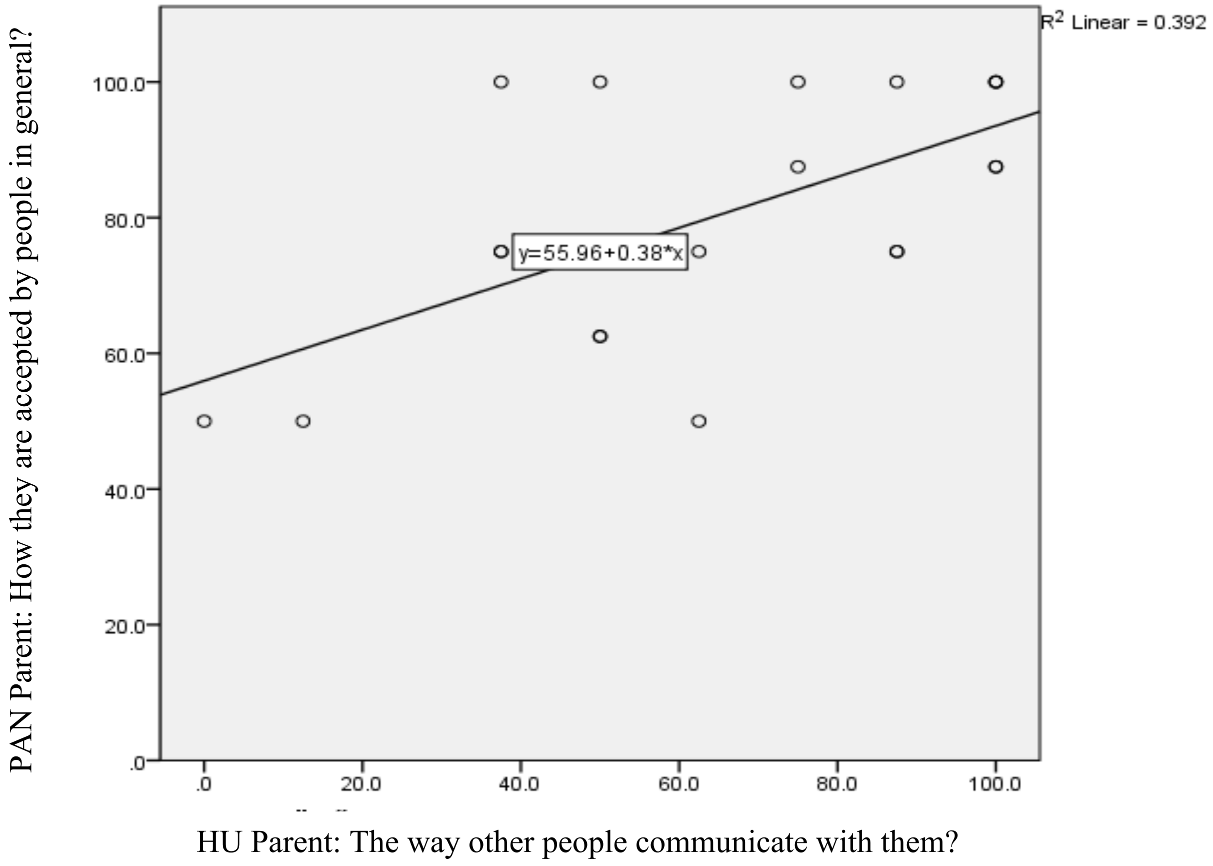
R2 Linear = 0.392
y=55.96+0.38*x
100.0
80.0
60.0
40.0
20.0
.0
.0
20.0
40.0
60.0
80.0
100.0
PAN Parent: How they are accepted by people in general?
HU Parent: The way other people communicate with them?

PAN Parent: Their positive attitude?

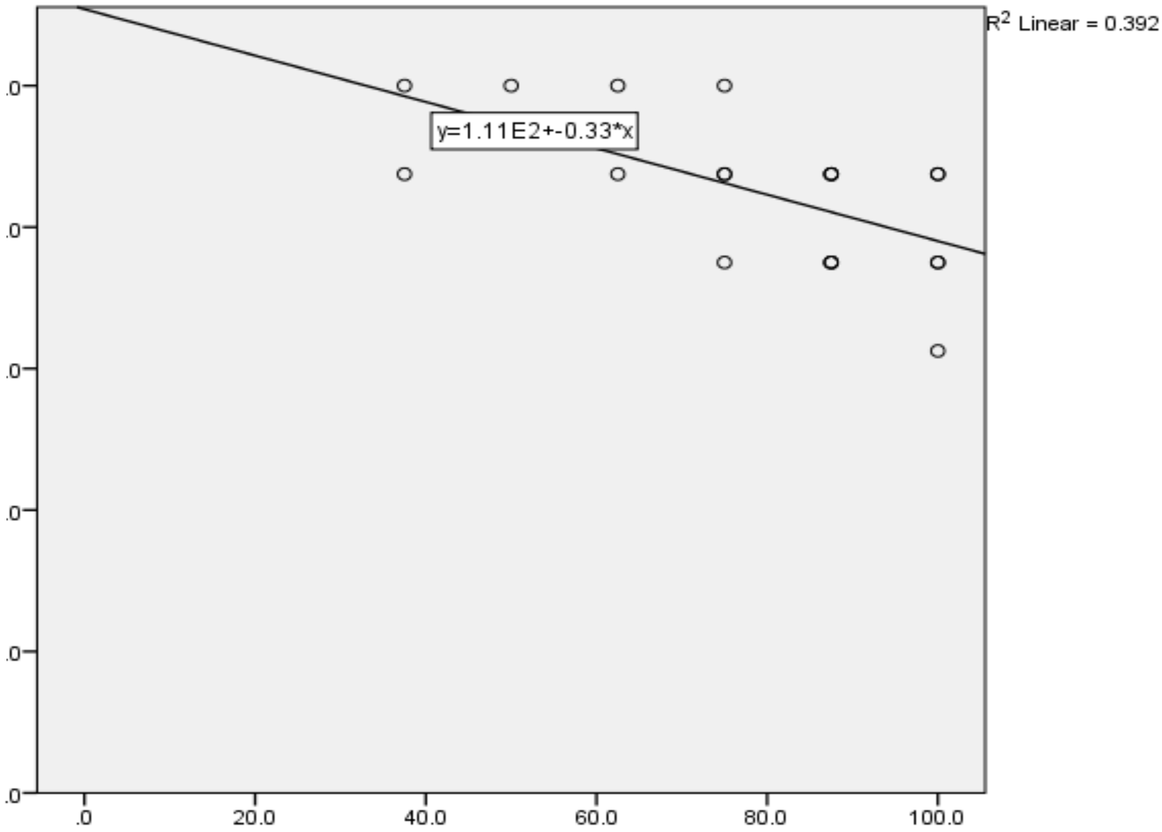


HU Parent: Their life in general??
